# Climatic variables and malaria transmission dynamics in Jimma town, South West Ethiopia

**DOI:** 10.1186/1756-3305-4-30

**Published:** 2011-03-02

**Authors:** Abebe Alemu, Gemeda Abebe, Wondewossen Tsegaye, Lemu Golassa

**Affiliations:** 1 Department of Medical Laboratory Sciences and Pathology, Jimma University, Jimma, Ethiopia; 2 Department of Medical Laboratory Sciences, College of Medicine and Health Sciences University of Gondar, Gondar, Ethiopia; 3 Department of Epidemiology and Social Medicine, University of Antwerp, Antwerp, Belgium

## Abstract

**Background:-:**

In Ethiopia, malaria is seasonal and unstable, causing frequent epidemics. It usually occurs at altitudes < 2,000 m above sea level. Occasionally, transmission of malaria occurs in areas previously free of malaria, including areas > 2,000 m above sea level. For transmission of malaria parasite, climatic factors are important determinants as well as non-climatic factors that can negate climatic influences. Indeed, there is a scarcity of information on the correlation between climatic variability and malaria transmission risk in Ethiopia in general and in the study area in particular. Therefore, the aim of this study was to determine the level of correlation between meteorological variables and malaria cases.

**Methods: -:**

Time-series analysis was conducted using data on monthly meteorological variables and monthly total malaria in Jimma town, south west Ethiopia, for the period 2000-2009. All the data were entered and analyzed using SPSS-15 database program. Spearman correlation and linear regression analysis were used to asses association between the variables.

**Results: -:**

During last ten years (2000-2009), a fluctuating trend of malaria transmission was observed with *P.vivax *becoming predominant species. Spearman correlation analysis showed that monthly minimum temperature, total rainfall and two measures of relative humidity were positively related with malaria but monthly maximum temperature negatively related. Also regression analysis suggested that monthly minimum (p = 0.008), monthly maximum temperature (p = 0.013) and monthly total rainfall (p = 0.040), at one month lagged effect, were significant meteorological factors for transmission of malaria in the study area.

**Conclusion: -:**

Malaria incidences in the last decade seem to have a significant association with meteorological variables. In future, prospective and multidisciplinary cooperative research involving researchers from the fields of parasitology, epidemiology, botany, agriculture and climatology is necessary to identify the real effect of meteorological factors on vector- borne diseases like malaria.

## Background

Malaria is caused by protozoan parasites of the genus *Plasmodium*. It is one of the leading causes of illness and death in the world. Nine out of ten of these deaths occur in Africa and the rest occur in Asia and Latin America, being the world's most prevalent vector-borne disease. It is the fourth leading cause of death of children under the age of five years and pregnant women in developing countries [1,2]. Also, the disease remains one of the most important causes of human morbidity and mortality with enormous medical, economic and emotional impact in the world. More than half of the world's population is at risk of acquiring malaria, and the proportion increases each year because of deteriorating health systems, growing drug and insecticide resistance, climate change and natural disasters [3,4].

Malaria is a leading public health problem in Ethiopia. It is estimated that about 75% of the total area of the country and 65% of the population is at risk of infection. *P.falciparum *and *P.vivax *are the main two species accounting for 60% and 40% of malaria cases respectively [5-7]. *Anopheles arabiensis *is the major malaria vector followed by *An. pharoensis *and other secondary vectors include *An.funestus *and *An. nili *[7,8]. Like all other mosquitoes, these breed in water, each species having its preferred breeding grounds, feeding patterns and resting place. Their sensitivity to insecticides is also highly variable [9].

Meteorological factors are important drivers of malaria transmission by affecting both malaria parasites and vectors directly or indirectly. Temperature, rainfall and humidity have been associated with the dynamics of malaria vector populations and, therefore, with spread of the disease. Especially, ambient temperature plays a major role in the life cycle of the malaria vector. The development of the parasite within the mosquito (sporogonic cycle) is also dependent on temperature. It takes about 9 to 10 days at temperatures of 28°C, but stops at temperatures below 16°C. The minimum temperature for parasite development of *P. falciparum *and *P.vivax *approximates 18°C and 15°C, respectively [10]. Also the daily survival of the vector is dependent on temperature. At temperatures between 16°C and 36°C, the daily survival is about 90%. The highest proportion of vectors surviving the incubation period is observed at temperatures between 28°C - 32°C [10,11]. Rainfall provides breeding sites for mosquitoes to lay their eggs, and ensures a suitable relative humidity of at least 50 to 60% to prolong mosquito survival [12].

Changes in temperature, rainfall, and relative humidity due to climate change are expected to influence malaria directly by modifying the behavior and geographical distribution of malaria vectors and by changing the length of the life cycle of the parasite. Climate change is also expected to affect malaria indirectly by changing ecological relationships that are important to the organisms involved in malaria transmission (the vector, parasite, and host). Recent evidence shows that changes in temperature and precipitation have already changed the distribution and behavior of the vector [13]. A study in Ethiopia had reported that although an epidemic was associated with higher rainfall [14], an epidemic in another year was preceded by very little rain. A reduction in malaria infection occurred in the Usambara Mountains of Tanzania following 2.4 times more rainfall than normal [15], while excessive rainfall during the same period was associated with increased malaria in south-western highlands of Uganda [16]. More over, another study found that variation in the relationship between the mosquito population and rainfall in different districts of Kenya and attributed the variation to environmental heterogeneity [17]. Similarly, other studies in the East African highlands showed there was high spatial variation in the sensitivity of malaria outpatient numbers to climate fluctuations [18]. In line with these, different studies in different parts of Africa and other continents concluded that at one month lagged effect meteorological variables are more likely correlated and predicts malaria cases occurrence than at zero month effect. They also reported that one meteorological variable might be more likely correlated than other meteorological variables [19-24].

In Ethiopia, the epidemiological pattern of malaria transmission is generally unstable and seasonal, the level of transmission varying from place to place because of differences in altitude and rainfall patterns. Changes have been observed in the epidemiology of malaria through time. Previously, malaria was known to occur in areas below 2000 m but currently it has been documented to occur indigenously even in areas above 2400 m, such as Addis Ababa, Akaki [25]. So, unstable malaria occurs in most parts of the country particularly in the highland fringes where climatic conditions are conducive for its transmission. The major transmission of malaria follows the June to September rains and occurs between September to December while the minor transmission season occurs between April to May following the February to March rains. Some localities also experience perennial malaria, because the environmental and climatological situations permit the continual breeding of vectors in permanent breeding sites.

Many time-series studies and studies of epidemics have been done to find explanatory variables for changes in malaria transmission, but many of them fail to take climatic factors into an account. Those studies mainly reported the relationships between factors other than climate that affect malaria rates such as urbanization, migration, irrigation, agricultural practices, deforestation, and malaria control efforts. This makes it difficult to assess the true determinants of malaria in given area. Also there is a lack of understanding of the complex interactions between meteorological variability and malaria infections or transmission risk in Ethiopia. In the study area, the link between meteorological factors, variability due to climate change and malaria transmission risk was not studied despite increased occurrence of malaria. This study was, therefore, initiated to explore any patterns of correlation exist between meteorological factors and malaria case occurrence over the last decade.

## Methods

### Study area

The study was conducted at Jimma town which is located 350Km south-west of Addis Ababa. The town's geographical coordinates are approximately 7°41' N latitude and 36° 50'E longitude. The town is found in an area of average altitude of about 1780 m above sea level. It lies in the climatic zone locally known as Woyna Daga which is considered ideal for agriculture as well as human settlement. The town is generally characterized by warm climate with a mean annual maximum temperature of 30°C and a mean annual minimum temperature of 14°C. The annual rainfall ranges from 1138 mm to 1690 mm. Maximum precipitation occurs during the three months period, June to August, with minimum rainfall in December and January. From a climatic point of view, abundant rainfall makes this region one of the best watered of Ethiopian highland areas, conducive for agricultural production.

### Study design

A retrospective study was conducted to determine the correlation between meteorological variables and malaria case occurrence over last decade in study area.

### Blood film preparation and examination of malaria parasites

Peripheral smear examination of a well-prepared and well-stained blood film is the gold standard in confirming the presence of the malaria parasite. In Ethiopia, the staining techniques and blood film examination for malaria parasite detection were conducted according to a standard operating procedure (SOP) in each health center through the country. In brief, before collection the finger was cleansed with an alcohol-moistened swab, dried with a piece of dry cotton, punctured with a disposable blood lancet. After wiping off the first drop of blood, thick and thin films were made on the same slide. After being air-dried in a horizontal position, the thin blood films were fixed in methanol for 30 sec. Then smears were stained with 10% Giemsa solution for 20 min. Each slide was examined under an oil immersion microscopic objective by experienced laboratory technicians or technologists who were certified in malaria diagnosis and species identification by the Ethiopian Ministry of Health. One hundred fields were examined before a negative result was reported. The thick smear was used to detect malaria parasites and parasite quantification. Then the thin smear was used to identify the *Plasmodium *species.

These microscopically confirmed malaria cases were monthly reported to zonal health office which was again reported to the Ethiopian Ministry of Health. Therefore, for this study, ten years (2000-2009) monthly malaria cases series data were obtained from Jimma town/zone heath offices which were reported from health centers, clinics and hospitals.

### Meteorological data collection

After receiving permission from the Meteorology agency/office, the previous 10 years (2000- 2009) monthly minimum, maximum and mean temperature, total rainfall and relative humidity of the town was obtained.

### Non-climatic factors affecting malaria cases occurrence

During malaria data collection, any malaria intervention activities that had been taken in each year to control malaria were collected using a well-prepared checklist from different responsible offices/ agencies or individuals in order to minimize the confounders that can negate meteorological influences. There was increased attention to malaria control and preventive activities by different responsible bodies, increased awareness of the community on use of insecticide treated bed nets (ITNs) and other malaria control activities through health education, increased accessibility of ITNs to community, increment of budget for malaria control and prevention activities (personal communication). But in the study area there were no special factors that attribute the increased or decreased occurrence of malaria cases.

Again, other factors like antimalarial drug resistance of the study area were obtained from Hinari and Enterz-PubMed web site and through personal communication. In the study area, chloroquine was the most abundant and commonly used antimalarial drug until resistance was reported. In Ethiopia, the increased resistance of *P. falciparum *to chloroquine (CQ) and sulfadoxine-pyrimethamine (SP) necessitated a change as first-line antimalarial drug for the treatment of *P. falciparum*. Consequently, Artemether/Lumefantrine (Coartem^®^) (AL) was adopted in 2004 but it became available in all regions of the country in 2005 [27-29]. Currently the common drug used for treatment of *P. vivax *malaria in the study area and throughout country is chloroquine because data on chloroquine resistance to *P.vivax *malaria is not sufficient enough to warrant a change. Again, in the study area *P.vivax *is the common malaria species and self treatment is also common, so there is a danger that *P.vivax *has developed or will develop the resistance to chloroquine.

### Data analysis

Average yearly mean temperature, total rainfall and relative humidity (January through December for each year) were calculated. All data from meteorological and clinical records were checked for completeness and cleaned of any inconsistencies. The data were entered to SPSS version 15.0 statistical software (SPSS Inc. Chicago, 2007).

To observe the correlation between meteorological variables and malaria cases, the monthly malaria cases were regarded as the dependent variables, while meteorological variables such as monthly maximum, minimum and mean temperature, total monthly rainfall and monthly relative humidity were independent variables. Spearman correlation analysis was conducted to examine the type and strength of relationship between meteorological variables and malaria cases. Then to observe independent effect of each independent variable on outcome, variable linear regression was fitted. Since there might be auto-correlation among independent variables over time, autocorrelation analysis was conducted. When the correlation coefficient for the association between these independent variables was larger than 0.5, these variables were analyzed in different regression model to reduce multicollinearity. The distribution of malaria and meteorological data was examined and all were approximately normally distributed.

## Results

### Annual trends of malaria cases in Jimma town, 2000-2009

**Figure 1:**
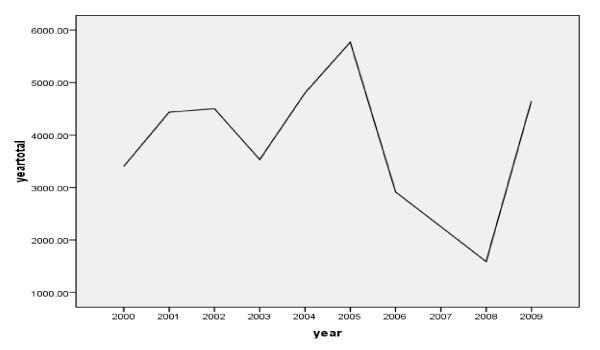
**Annual trends of total malaria cases in Jimma town, 2000-2009**. The figure shows that an increase in malaria cases occurrence from 2003-2005 with peak cases occurring in 2005 and malaria cases were reduced the following three consecutive years (2006-2008) but a remarkable increase in 2009.

A fluctuating trend of malaria cases reported through the years 2000 to 2009 was observed. An increase in malaria cases occurrence from 2003-2005 with peak cases occurring in 2005 and malaria cases were reduced the following three consecutive years (2006-2008) but a remarkable increase in 2009 was observed. During the last ten years, a total of 37,862 microscopically confirmed malaria cases were reported in the town with the annual total cases of malaria ranged from 1590 in 2008 to 5860 in 2005 with 3759 mean annual malaria cases occurring. There was statistically significant inter- annual variation of malaria cases occurrence in the study area (p = 0.007) (Figure 1).

**Figure 2:**
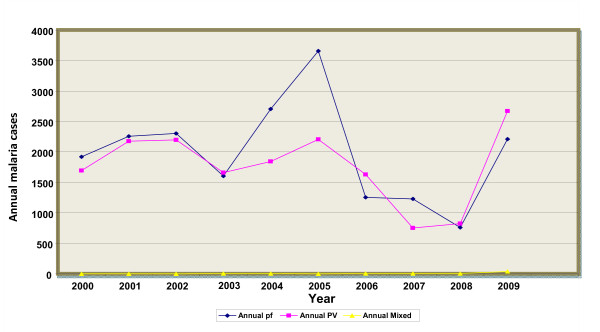
**Relative annual trends of *P. falciparum *and *P. vivax *malaria in Jimma town, 2000-2009**. The figure demonstrates that a high fluctuation of malaria cases at species level in the study area. With the exception of some years, a high predominance of *P. falciparum *over *P. vivax *was observed. Thus the remarkable increment in total malaria cases was mainly due to the increment of *P. falciparum *rather than *P. vivax *except that by the year 2009 *P. vivax *was found to be highly diagnosed relative to *P. falciparum *which indicates that currently there was a trend shift from *P. falciparum *to *P. vivax *malaria in the study area.

A high fluctuation of malaria cases at species level was also observed with statistically significant (p < 0.001) inter- annual variation of both *P.vivax *and *P.falciparm *malaria cases occurrence. With the exception of some years, a high predominance of *P. falciparum *over *P. vivax *was observed within the last ten years. Thus the remarkable increment in total malaria cases was mainly due to the increment of *P. falciparum *rather than *P. vivax *except that by the year 2009 *P. vivax *was found to be highly diagnosed relative to *P. falciparum *which indicates that currently there was a trend shift from *P. falciparum *to *P. vivax *malaria in the study area (Figure 2).

### Monthly and seasonal variation of malaria cases in Jimma town, 2000-2009

**Figure 3:**
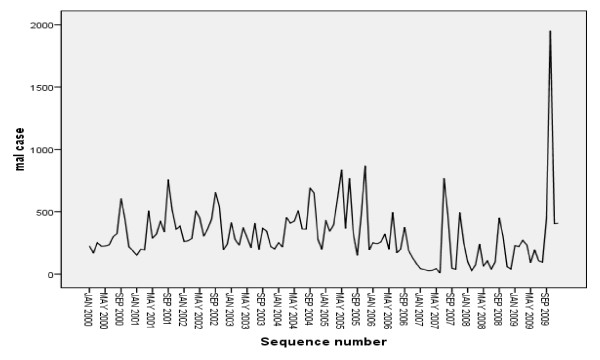
**Monthly and seasonal variation of total malaria cases in Jimma town, 2000-2009**. The figure indicates that in the study area malaria cases occurred in almost every month of the year. The highest peak of malaria cases in almost all years was observed during September with exception that in 2009 the highest cases were observed during October. Again the season with the highest average total malaria cases occurrence was spring (September, October and November ) and the minimum malaria cases was observed during winter (December, January and February ).

Despite the apparent fluctuation of malaria trends in the study area malaria cases occurred in almost every month of the year. The highest peak of malaria cases in almost all years was observed during September with exception that in 2009 the highest cases were observed during October. There was statistically significant variation of monthly malaria cases occurrence (p = 0.036) (Figure 3). The season with the highest average total malaria cases occurrence was spring (September, October and November ) and the minimum malaria cases was observed during winter (December, January and February ). For total malaria cases, the seasonal variation was statistically significant (p = 0.007). At species level, both *P.vivax *and *P.falciparm *maximum cases were observed in spring followed by autumn ( March, April and May ) and the minimum being during winter followed by summer (June, July and August). In all seasons *P. falciparum *cases were higher than *P. vivax*. In years of high malaria cases, the spring peak was more pronounced when compared with other years and there was a substantial number of cases late in the year.

### Trends of meteorological factor variations in Jimma town, 2000-2009

Unlike malaria cases, from 2000- 2009 there was no statistically significant inter- annual variations of all measured meteorological factors (temperature, rainfall and relative humidity) in the study area. But there were statistically significant (P < 0.001) inter- monthly and inter- seasonal variations among those measured meteorological variables.

**Figure 4:**
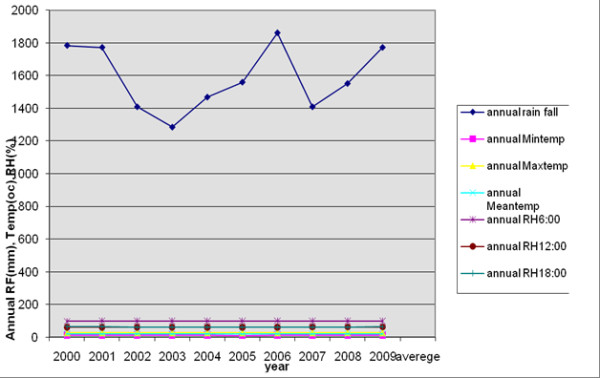
**Annual meteorological factors variation in Jimma town, 2000 -2009**. The figure demonstrates that in the town, annual mean temperature ranged from as low as 19.5°C in 2005 to as high as 20.1°C in 2009 and a slight fluctuating trend of temperature through the years 2000 to 2009 was observed. It also shows a high fluctuating trend of rainfall was reported through the years 2000 to 2009 with 1586.7 mm mean annual rainfall and maximum total rainfall was observed in 2006 (1859.9 mm) and the minimum rainfall was observed in 2003 (1285.2 mm). In the last ten years, a fluctuating trend of relative humidity was observed at three different hours (0600, 1200 and 1800).

The Jimma town, annual mean temperature ranged from as low as 19.5°C in 2005 to as high as 20.1°C in 2009 and a slight fluctuating trend of temperature through the years 2000 to 2009 was observed. But a high fluctuating trend of rainfall was reported through the years 2000 to 2009 with 1586.7 mm mean annual rainfall and maximum total rainfall was observed in 2006 (1859.9 mm) and the minimum rainfall was observed in 2003 (1285.2 mm). In the last ten years, a fluctuating trend of relative humidity was observed at three different hours (0600, 1200 and 1800). The average relative humidity at three different hours ranged from 59.3% to 85% in the last ten years (Figure 4).

### Correlation between malaria cases and meteorological variables

**Figure 5:**
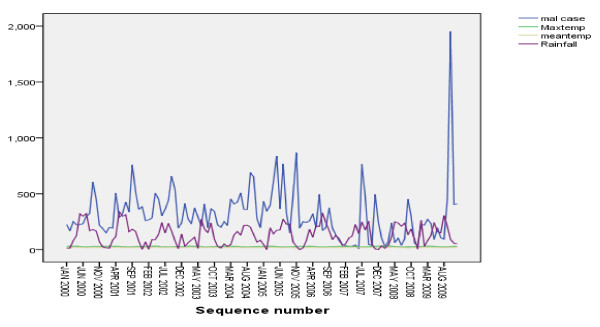
**Relationship between monthly malaria cases and meteorological variables in Jimma town, 2000-2009**. The figure demonstrates association between monthly malaria cases and meteorological variables (temperature, rainfall and relative humidity) in the same graph. There was fluctuation of malaria cases with rainfall fluctuation in the figure. However, other meteorological variables were less likely fluctuates.

An association between monthly malaria cases and meteorological variables (temperature, rainfall and relative humidity) was observed. However, there was high simple relationship between monthly total rain fall and monthly total malaria cases than other meteorological variables in the study area (Figure 5). These relationships among malaria and meteorological variables were father checked by Spearman's correlation and linear regression analyses.

Spearman's correlation analyses were conducted relating both monthly total and each species of malaria cases to various meteorological measures (maximum temperature, minimum temperature, mean temperature, total rainfall, relative humidity at 06:00, 12:00 and 18:00 and average relative humidity) at both a zero month effect and one month lagged effect. At zero month effect, none of meteorological variables was statistically significantly correlated with malaria. Monthly mean and maximum temperature were negatively correlated with monthly total malaria cases occurrence but other variables were positively related (Table 1).

**Table 1:** Correlation between meteorological variables and monthly total malaria cases, at zero month effect, Jimma town, 2000-2009

Meteorological variables	Correlation coefficient	P. value
Maximum temperature	-0.171	0.062
Minimum temperature	0.143	0.120
Mean temperature	-0.024	0.135
Rainfall	0.122	0.092
Relative humidity at 6 am	0.024	0.074
Relative humidity at 12 am	0.122	0.075
Relative humidity at 18 pm	0.140	0.064
Average relative humidity	0.120	0.068

Unlike the relation between total monthly malaria cases occurrence and meteorological variables at zero month effect, there was statistically significant (p = 0.027) positive correlation between monthly *P.vivax *case occurrence and minimum temperature and statistically significant (p = 0.014) negative correlation between monthly *P.vivax *case occurrence and maximum temperature. But none of meteorological variables were statistically significantly correlated with monthly *P. falciparum *case occurrence.

Monthly maximum temperature and relative humidity measured at 06:00 were negatively correlated with monthly total malaria cases occurrence with one month lagged effect but others factors were positively related. Unlike the zero month relationship there were statistically significant positive correlations between total malaria cases occurrence and total rainfall, relative humidity at 12:00, relative humidity at 18:00 and minimum temperature. There was, however, statistically significant negative correlation between monthly total malaria cases occurrence and maximum temperature with one month lagged effect (Table 2).

**Table 2:** Correlation between meteorological variables and monthly total malaria cases, one month lagged effect in Jimma town, 2000-2009

Meteorological variables	Correlation coefficient	P. value
Maximum temperature P1 *	-0.200	0.029
Minimum temperature P1 *	0.223	0.015
Mean temperature P1 *	0.037	0.689
Rainfall P1 *	0.214	0.020
Relative humidity at 6 am P1 *	-0.045	0.628
Relative humidity at12 am P1 *	0.191	0.038
Relative humidity at 18 pm P1 *	0.180	0.049
Average relative humidity P1 *	0.177	0.054

* Prior month

Autocorrelation among the independent variables was investigated (Table 3). There were high positive correlations between monthly total rainfall with two measures of relative humidity (at 12:00 and 18:00). Because the correlation coefficient for the association between these independent variables was larger than 0.6, these variables were analyzed in a different regression model to reduce multicollinearity.

**Table 3:** Autocorrelation among meteorological variables in Jimma town, 2000-2009

Variables	Max temp	Min temp	Mean temp	Rainfall	RH 6 am	RH12 am	RH18 pm
Max temp	1.000	-.651	.391	-.638	-.002	-.786	-.801
Min temp	-.651	1.000	.357	.786	-.251	.835	.757
Mean temp	.391	.357	1.000	.218	-.200	.054	-.013
Rainfall	-.638	.786	.218	1.000	-.066	.805	.787
RH 6:00	-.002	-.251	-.200	-.066	1.000	-.089	-.010
RH12:00	-.786	.835	.054	.805	-.089	1.000	.911
RH18:00	-.801	.757	-.013	.787	-.010	.911	1.000

To observe independent effects of each independent variable on malaria cases occurrence, linear regression analysis was conducted. At zero month effect from all meteorological factors by simple linear regression, maximum temperature was the only statistically significant (p = 0.032) meteorological factor for monthly total malaria cases. But when all meteorological factors were entered in multi- linear regression the model was statistically insignificant (p = 0.129) and the variables were not likely to yield equations or models that are useful for predicting malaria cases occurrence in the study area.

At one month lagged effect, monthly minimum and maximum temperature and monthly total rainfall were statistically significant (p = 0.036) meteorological factors for monthly total malaria case occurrence/transmission in Jimma town. At individual levels and together these meteorological variables gave small beta (correlation coefficient) and small R square value (the coefficient of determination) (Table 4).

**Table 4:** Simple Linear regression for monthly total malaria cases occurrence and meteorological variables at one month lagged effect, Jimma town, 2000-2009

Variables	Beta	R square	P- value
Maximum temperature P1 *	-0.227	0.052	0.013
Minimum temperature P1*	0.241	0.05	0.008
Total rainfall P1 *	0.068	0.036	0.040

* Prior month

## Discussion

The transmission of malaria can be determined by climatic or non- climatic factors. But even leaving these non-climatic issues aside, the effect of climate itself on the intrinsic probability of malaria transmission, remains controversial. So, climate variability that impacts on the incubation rate of *Plasmodium *and breeding activities of *Anopheles *is considered one of the important environmental contributors to malaria transmission dynamics [13,26].

For example, temperature rise is expected to increase transmission and prevalence of malaria by reducing the interval between mosquito blood meals, thus decreasing the time to produce new generations and by shortening the incubation period of the parasite in the mosquitoes. Sporogonic cycles take about 9 to 10 days at temperatures of 28°C but higher than 30°c and below 16°C have negative impact on parasite development [10]. Also the minimum temperature for *P. falciparum *and *P.vivax *parasite development approximates to 18°C and 15°C, respectively and the daily survival of the vector is dependent on temperature as well. At temperatures between 16°C and 36°C, the daily survival is about 90%. The highest proportion of vectors surviving the incubation period is observed at temperatures between 28° - 32°C. So, temperature of 20°C to 30°C and relative humidity greater than 60% are optimal for *Anopheles *survive long enough to acquire and transmit the parasite [10].

The result of our study revealed that during the last ten years, a fluctuating trend of occurrence of malaria cases was observed in Jimma town. An increase in malaria cases occurrence from 2003-2005 with peak cases occurring in 2005 and malaria cases were reduced the following three consecutive years (2006-2008) but a remarkable increase in 2009 was observed. Except for the year 2009, the remarkable increment of total malaria cases was mainly due to an increase of *P.falciparum *with little increase of *P.vivax*. But for 2009 total malaria cases, *P.vivax *contributed more than *P.falciparum*. Resistance of *P. falciparum *to the commonly used drug (chloroquine) during these years may have contributed to total malaria case occurrence [27-29]. In those years with remarkable malaria increase, from all meteorological variables only annual rainfall was increased and showed a positive relationship but it was not statistically significant (p = 0.069).

The occurrence of malaria was reduced during three consecutive years from 2006-2008. It coincides with the increased availability of the new drug Coartem for *P.falciparum *malaria at national and local level. A decrease in malaria cases occurrence after the 2005 maximum occurrence was observed. The increased attention to malaria control and preventive activities by different responsible bodies, increased awareness of the community on use of ITNs and other malaria control activities, increased accessibility of ITNs to community, increment of budget for malaria control and prevention activities (personal communication and data not shown) might contributed the decrement in malaria case occurrence in addition to meteorological factors.

It is likely that the excessive flooding due to heavy rains in 2006 might have also impaired mosquito breeding and flushed out the mosquito larvae [12]. From all the months, the highest monthly malaria cases occurrence was observed during October in 2009 which was the main contributor for 2009 annual malaria cases increase. In this month a small increase of minimum temperature during September after summer rainfall, because correlation analysis at one month lagged effect minimum temperature, was the first meteorological variable that significantly affects malaria transmission in the study area. Some other non-climatic factors, such as road constructions and some other activities in the town which increased the number of breeding sites of mosquitoes might have contributed to the peak malaria occurrence (personal observation) or it might be due to increase of temperature from 11.7°C in 2008 to 12.2°C in 2009 or annual rainfall was increased from 1551 mm in 2008 to 1770.9 mm in 2009 or might be due to resistance of *P. vivax *for currently available drug (chloroquine) in the market [30,31].

According to correlation findings, monthly maximum and mean temperature at zero month effect and maximum temperature and relative humidity at o6oo at one month lagged effect were negatively correlated but other meteorological factors were positively related with total monthly malaria case occurrence. The finding implies that meteorological variables can affect malaria transmission either positively or negatively even if the correlation was less likely linear. This finding contradicts with the findings in Shuchen County, China [20] and Highlands of Madagascar [22] which showed that all meteorological variables were positively correlated with malaria. The present study was undertaken at different altitude in Jimma. Correlation between malaria and climate vary with altitudes [13]. The correlation coefficient for the association between monthly malaria cases and some meteorological factors was greater than other meteorological factors. This indicates that one meteorological factor plays greater role in malaria cases occurrence or transmission than others which coincide with the finding from Dehradun, Uttaranchal, India [19] Shuchen county, China [20], Rwanda [21], Madagascar [22] and east Africa Highlands [23].

In this study, the correlation coefficient for the association between monthly mean minimum temperature and monthly malaria cases was greater than that of the correlation coefficient for the association between any other measured meteorological variables and monthly malaria cases. Our results suggest that mean minimum temperature was the most significant factor that correlated with malaria transmission dynamics in the study area. The results of a similar study conducted in Rwanda suggested that monthly malaria cases occurrence or incidence in high altitude regions is related to change in minimum temperature, while in low altitude zone rainfall and mean minimum temperature was the most significant meteorological factor [21]. It is also similar to the findings in Madagascar [22]; China [20] and east African Highlands [23]; all suggested that minimum temperature was most significant factor for malaria transmission over other meteorological factors. Therefore a rise of temperature, especially minimum temperature, would enhance the survival of *Plasmodium *and *Anopheles *during different seasons and thus accelerate the transmission dynamics of malaria and spread it into populations that are currently malaria free and immunologically naïve.

The monthly total rainfall was the most significant factor that determines malaria transmission in the study area after to minimum temperature. Rainfall plays an important role in malaria epidemiology because water not only provides the medium for the aquatic stage of the mosquitoes' life cycle but also increases the relative humidity and then the longevity of the adult mosquitoes. In some Sub-Saharan countries, for example, malaria transmission is restricted largely to the rainy seasons [13]. However, the effect of rainfall on the transmission of malaria is very complicated, varying with the circumstances of particular geographical regions and depending on the local habits of mosquitoes. Rainfall may prove beneficial to mosquito breeding if moderate, but it may destroy breeding sites and flush out the mosquito larvae when it is excessive [12]. This study indicates that total monthly rainfall was associated with occurrence of malaria in the town with a month lag effect. The same results can also found in Shucher County, China [20] and New Halfa, Eastern Sudan [24].

On the other hand, the correlation coefficients for the linear regression between the monthly mean and maximum temperature and monthly malaria cases were negative. This finding was similar to a study in India [19]. This is important in the hot months, in which an increase in temperature would limit vector and parasite survival and therefore cause a decrease in malaria transmission rates. This finding contradicts which the findings in Shucher County, China which concluded that an increase in monthly maximum temperature should cause an increase rather than a decrease in malaria rates [20]. This variation could be due to differences in local climatic condition in China and Jimma. That is the large number of months in Jimma that are hotter than months in China - this makes sense in the hot months, in which an increase in temperature would limit vector and parasite survival and therefore may cause a decrease in malaria transmission rate. The most likely explanation for the finding that increases in temperatures is correlated with a decrease in malaria cases is the significant autocorrelation between monthly temperature and relative humidity. This hypothesis is supported by the finding of high negative correlation between temperature and relative humidity. This indicates that, for a given amount of moisture in air, an increase in temperature cause a decrease in relative humidity, which can limit *Anopheles *survival. The correlation between maximum temperature and rainfall may also lead to an explanation of the negative correlation coefficient between maximum temperature and malaria cases occurrence. The negative correlation between maximum temperature and rainfall in hotter months may decrease *Anopheles *breeding or increase dryness which may be the limiting factor for malaria transmission.

Both the correlation and regression analyses suggests that temperature, rainfall and relative humidity act on monthly malaria case total occurrence with a lag of one month. Although all meteorological variables were less likely to predicts the occurrence of malaria in Jimma town. This finding contradicts the findings in Dehradun, Uttaranchal, India [19] Shuchen County, China [20], Rwanda [21], Madagascar [22] and east Africa Highlands [23] which concluded that at one month lagged effect meteorological variables were highly likely correlated with malaria occurrence and the prediction was higher than this finding with higher R square value. This variation might be due to the fact that this study was conducted in lowlands in which malaria is endemic. In lowlands, the factors that contribute to malaria transmission dynamics are microclimate variation due to anthropogenic effects and other non- climatic factors like, health system, population growth, population movement and others [13,26].

At zero month time effect none of meteorological variables were statistically significantly correlated with monthly *P.falciparm *cases occurrence and total monthly malaria cases but there were statistically significant positive correlation between monthly *P.vivax *cases and minimum temperature and statistically significant negative correlation between monthly *P.vivax *case occurrence and maximum temperature. This might be due to the fact that *P.vivax *requires a little lower temperature than *P.falciparum *[11]. Thus, minimum temperature variability could have more effect on *P. vivax *than *P.falciparm *by shortening the extrinsic phase and little change in maximum temperature have more negative effect on the development of *P.vivax *than *P. falciparum*.

Seasonality and year (time trend) played a role in the transmission of malaria in the town. From the time series analysis, none of the measured meteorological variables were able to predict malaria transmission dynamics. In general, there was a fluctuation in malaria cases during the last ten years. Many factors might be responsible for seasonal changes, e.g., climatic variables, ecologic and environmental factors, host and vector characteristics, and social and economic determinants such as change in health care infrastructure. Thus the range of vector borne disease is not solely determined by meteorological variability [32-35]. Social, biological and economic factors such as mosquito control measures, population immunity, local ecological environment (vegetation, introduction of irrigation ), governmental policy, availability of health facilitates and drug resistance have also an impact of malaria transmission dynamics. Also in the study there were different malaria control activities in each year like insecticide spraying, elimination of mosquito breeding sites, health education about malaria, distribution of ITNs and some malaria drugs and other activities to decrease mortality and morbidity of malaria. A limitation of the study was data on some of non- climatic factors which were available in the study area from annual reports but due to its incompleteness it was not included in regression analysis.

## Conclusion

Even though the result of trend analysis found that meteorological variables were less likely linearly correlated and predicts malaria cases occurrence in the study area, the changes in the current month meteorological factors would have effects on next month malaria case occurrence that provides an early warning which enables the health care system to be well prepared and to allocate scarce resource effectively to reduce mortality and morbidity of malaria. In the study area to decrease morbidity and mortality of malaria more attention should be given to microclimate change due to anthropogenic effect and other non- climatic factors that affects malaria transmission dynamics rather than meteorological variability due to global warming or climate change. In general, transmission of malaria is very complicated and detailed ecological and epidemiological studies are still needed to assess the true local risk. Further studies that account for all possible confounding factors and that are done at a smaller spatial scale, will improve our understanding of which factors will most affects malaria transmission dynamics in the study area.

## Competing interests

The authors declare that they have no competing interests.

## Authors' contributions

Abebe Alemu conceived the study, undertook statistical analysis and drafted the manuscript. Wondewossen Tsegaye, Gemeda Abebe and Lemu Golassa initiated the study, made data available in collaboration with National Meteorological Agency and Jimma town health bureau and made major contributions to the study design and statistical analysis. All authors contributed to the writing of the manuscript and approved the submitted version of the manuscript.
